# Multidisciplinary Approach of a Male Case of Imported Malaria, HIV Chronic Infection, and Latent Syphilis

**DOI:** 10.3390/idr16060091

**Published:** 2024-11-27

**Authors:** Rebeca Eunice García-Mendiola, Maritza Micheli García-Lucas, Jennifer Morales-Vázquez, Raúl Adrián Cruz-Flores, Miguel Ángel Loyola-Cruz, Clemente Cruz-Cruz, Emilio Mariano Durán-Manuel, Enzo Vásquez-Jiménez, Graciela Castro-Escarpulli, María de Jesús Sánchez-Guzmán, Victor Hugo Gutiérrez-Muñoz, Iliana Alejandra Cortés-Ortíz, Misael González-Ibarra, Juan Carlos Bravata-Alcántara, Jesús Alejandro Pineda-Migranas, Estibeyesbo Said Plascencia-Nieto, Carlos Alberto Jiménez-Zamarripa, Erika Gómez-Zamora, Claudia Camelia Calzada-Mendoza, Juan Manuel Bello-López

**Affiliations:** 1 Clínica Especializada Condesa Iztapalapa, Mexico City 09730, Mexico; 2 Hospital Juárez de México, Mexico City 07760, Mexico; 3 Sección de Estudios de Posgrado, Escuela Superior de Medicina, Instituto Politécnico Nacional, Mexico City 11340, Mexico; 4 Escuela Nacional de Ciencias Biológicas, Instituto Politécnico Nacional, Mexico City 11340, Mexico; 5 Departmento de Dietología, Hospital Psiquiátrico “Dr. Samuel Ramírez Moreno”, Valle de Chalco Solidaridad 56619, Mexico

**Keywords:** migration, imported malaria, HIV chronic infection, syphilis, Mexico City

## Abstract

Background: The current economic and social crisis in Latin America has caused migration to the USA, bringing with it Public Health challenges due to the importation of various infectious diseases. Migrants, particularly those with chronic conditions, such as HIV infection and other sexually transmitted infections (STI), are at greater risk due to pharmacological interruption and access to medical care, so the timely detection of diseases acquired during their migration, such as malaria, is crucial to avoid health complications. Objective: To outline by a multidisciplinary approach (Infectology, Parasitology, Epidemiology, molecular Biology, Venereology, and Public Health) the diagnosis and management of a male case with malaria imported to Mexican territory, HIV chronic infection, and latent syphilis. Methods: A male migrant of Venezuelan nationality attended the Clínica Especializada Condesa Iztapalapa in Mexico City for health complications. A comprehensive analysis of laboratory and molecular tests was performed to confirm HIV infection. During the STI diagnostic algorithm, latent syphilis was detected and microscopic observation of blood smears revealed parasitic forms compatible with malaria. Standard and molecular tests were applied under the operational definition for malaria cases for identification, diagnosis, and treatment. Finally, study of clinical history and migration route by questioning for the investigation of the imported case was performed. Results: The immigrant was diagnosed with HIV chronic-stage infection with interrupted antiretroviral therapy (ART), latent syphilis, and malaria by *Plasmodium vivax*. The ART administered was chosen based on the possible drug interaction with antimalarials and genetic barrier to the HLA-B* allele. Finally, antimicrobial therapy against syphilis was penicillin. From the analysis of the migratory route, incubation time of imported malaria, and questioning, we speculated that the migrant acquired the *P. vivax* infection in Panama. Conclusions: This case highlights the complex health problems faced by migrants with HIV infection, particularly when they contract additional infections such as malaria during migration and highlights the need for comprehensive access to healthcare and ART, antimalarial and antimicrobial treatments to mitigate the health risks of this vulnerable population.

## 1. Introduction

The intensification of the socioeconomic crisis experienced in recent years by inhabitants of Latin American countries has forced millions of people to leave their countries of origin in search of better living conditions [1,2]. This social phenomenon brings with it several associated problems, including those that impact the health of the native population, for example, through the importation of multidrug-resistant pathogens [3,4,5]. Conversely, migrants may acquire infections during their journey in disease-endemic countries, putting their health at risk. Since Mexico is the country bordering the USA as the last in the chain of countries transited by migrants, Mexican healthcare services serve as attention centers for migrants who require health services, from care for women in labor to those who have acquired infectious diseases during their journey, with vector-borne diseases being one of the most important.

In a previous work by our working group, we have reported cases of malaria imported by *Plasmodium vivax* and *P. falciparum* from Latin American and African migrants into Mexico, where we discussed various implications, including the risk of importing strains of *Plasmodium* spp. resistant to antimalarials [6]. Nevertheless, another type of consequence associated with the migration of this population, sexually transmitted infections (STIs), has been omitted. Epidemiological studies of STIs in our country have shown that syphilis and gonorrhea continue to be a Public Health problem that requires immediate attention [7,8]. Migrants with STIs such as uncontrolled HIV infection, latent syphilis, and gonorrhea, can act as dispersers of pathogens that in recent years have gained global importance due to the emergence of multidrug-resistant strains [9,10,11].

Migrant health issues must, therefore, be addressed holistically, particularly for those with bacterial and viral STIs, such as HIV chronic infection and, recently, Mpox infection, and diseases acquired during migration such as malaria. This highlights the importance of universal access to antiretrovirals, antimalarials, and antimicrobials in this population and shows the challenge for Mexican health services to provide multidisciplinary and comprehensive care to detect diseases of diverse etiologies in a timely manner. This report presents a case of a patient of Venezuelan nationality, HIV chronic infection, with latent syphilis, who acquired malaria by *P. vivax* during his migration.

The comprehensive diagnosis of malaria and syphilis, as well as the continuation of HIV virological suppression through antiretroviral therapy (ART), allowed the migrant to continue his migration journey to the USA. This case highlights the health challenges faced by Latin American migrants with multiple infectious diseases of bacterial, viral, and parasitic origin and the impact on the health of people with HIV infection who are forced to discontinue ART. The need for universal access to antiretrovirals, antimicrobials, and antimalarials, along with comprehensive and multidisciplinary diagnosis for migrants is analyzed and discussed.

## 2. Materials and Methods

### 2.1. Case Presentation

**Figure 1:**
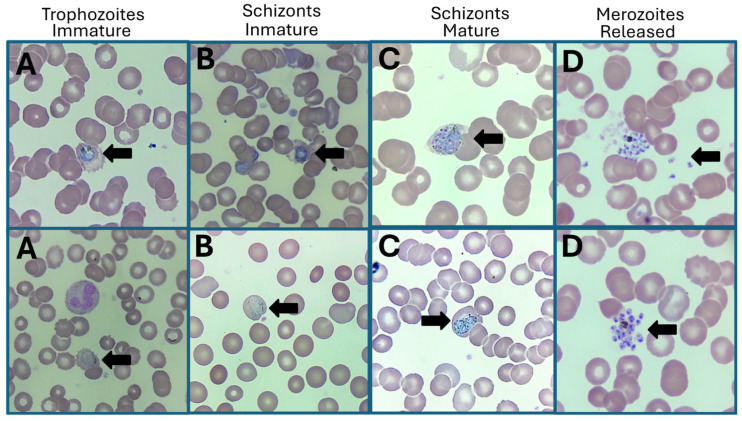
Detection of *P. vivax* by thick-drop test (Giemsa stain) in a male case with imported malaria, HIV chronic infection, and latent syphilis. Findings of parasitic stages (indicated by black arrows). (**A**) Trophozoites immature, (**B**) Schizonts immature, (**C**) Schizonts mature, and (**D**) Merozoites released. Magnification 1000×.

On 19 July 2024, a 27-year-old adult male patient of Venezuelan nationality attended the Clínica de Especialidades Condesa Iztapalapa, with diaphoresis, myalgia, chills, nausea, and fever of 40 °C. Because the patient was an HIV carrier with interrupted treatment per 33 days due to his migratory status (treatment shortage), he speculated that the health complication was due to the lack of ART, so medical attention was requested at this clinic, a medical center dedicated to the care of patients with STIs, including HIV infection. On interrogation, the patient indicated that he had been HIV-positive since March 2022, so a viral panel was performed by lateral flow immunochromatography, which was reactive for antibodies against HIV, and non-reactive for antigen HBsAg and antibodies against HCV. The rapid anti-TP test and RPR (1:32) for *Treponema pallidum* were positive, with no apparent lesions of primary or secondary syphilis. RT-PCR analysis of the viral load was performed, and the viral load was undetectable with a CD4+ cell count of 348 cells/mm^3^, classifying him as immunocompromised. He reported that he started having sex at the age of 14 with approximately 100 sexual partners (20 women and 80 men). He identified himself as bisexual with an active role and no history of sexual abuse. He indicated a history of contact with and practice of sex work, on one and five occasions, respectively. Liver function tests and lipid profile were within normal reference values. Outstanding findings on blood biometry were low hemoglobin levels (8.5 g/dL) and hematocrit of 26.7%. During Giemsa microscopic observation, anisocytosis (+++) and anisochromia (+) were detected, and structures specific to various stages of the *P. vivax* life cycle (immature trophozoites, immature schizonts, mature schizonts, and free merozoites) were detected. For this purpose, the best images with parasites isolated from erythrocytes were chosen to observe the different stages. Figure 1 shows the four stages of *P. vivax* detected (1000×) under thick-drop light microscopy in the imported malaria case of a patient with HIV chronic infection and latent syphilis. On questioning, the patient reported having been exposed to mosquito bites for approximately 30 days during his transit through Panama. However, there is a possibility that he had contact with anophelines in other countries along the migration route.

The integration of symptomatology, clinical, and laboratory findings led to the final diagnosis of “HIV chronic infection, normocytic anemia, latent syphilis of undetermined duration, and malaria by *P. vivax*”. In addition, on 29 July 2024, the Laboratorio de Vigilancia Epidemiológica de la CDMX de la Dirección de Epidemiología de la CDMX (Epidemiological Surveillance Laboratory of Mexico, City of the Epidemiology Directorate of Mexico, City) provided additional data on the case, such as positivity in the rapid test for the detection of *Plasmodium*-specific lactate dehydrogenase (PDR) and *P. vivax* parasitemia with 1188 parasites in the asexual stage/μL and 333 parasites in the sexual stage/μL. Thick-drop microscopy on three slides analyzed showed less than 10 parasites per field in a total of 500 fields analyzed, indicating a significant parasite load. Based on the information described above, the case fulfilled the necessary elements to be confirmed as imported malaria by *P. vivax*. Therefore, a formal epidemiological investigation of an imported malaria case was initiated in accordance with the General Directorate of Epidemiology and the manual of “operational definitions of diseases subject to surveillance” as follows.

### 2.2. Regulatory Policies in the Epidemiological Investigation of Imported Malaria Cases

Regulatory policies in Mexico establish mandatory reporting of new malaria cases in all 32 states of the country. Epidemiological data on new malaria cases are routinely deposited in the morbidity yearbooks available at www.epidemiologia.salud.gob.mx/anuario/html/index.html (accessed on 25 July 2024). The data reported on this website are analyzed and published the following year by the Ministry of Health, as incidence of cases (per 100,000 inhabitants). This case will be part of the malaria statistics on the SUAVEweb platform (www.sinave.gob.mx, accessed on 25 July 2024) by 2025.

### 2.3. Operational Definition of a Malaria Case

Mexico’s General Directorate of Epidemiology (DGE, by its acronym in Spanish) has the manual “operational definitions of diseases subject to surveillance”, which classifies malaria in the group of vector-borne diseases and subclassifies it into two types according to the infecting *Plasmodium* species [12]. The definition of a probable case is a person who resides in or comes from areas with a history of malaria, and who, in the last month, presents or has presented fever, headache, diaphoresis, and chills. In some cases, the clinical picture may vary and include sweating, cough, diarrhea, respiratory distress, jaundice (depending on the *Plasmodium* species), coagulation defects, shock, renal and hepatic failure, acute encephalopathy, pulmonary and cerebral edema, coma, and death. After a fever-free period, the cycle of chills, fever, and sweating repeats, every two to three days. Confirmation of a case is microscopic demonstration of trophozoites, schizonts, and/or gametocytes by Giemsa- or Wright-stained thick-drop test [12]. The manual recommends additional molecular tests, but they are not mandatory under Mexican regulations; however, to support the present case investigation, additional genetic evidence was generated as follows.

### 2.4. Genetic Confirmation of Plasmodium vivax in Imported Malaria

**Figure 2:**
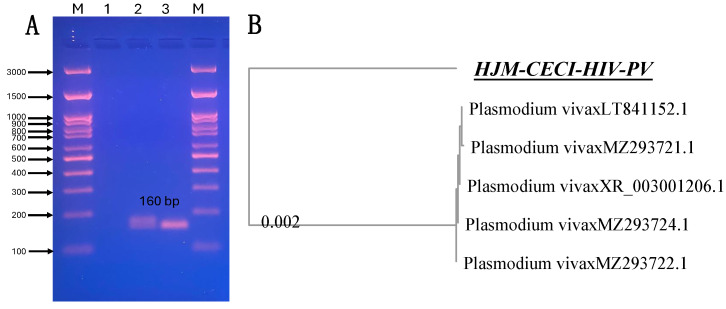
(**A**) Molecular detection of *P. vivax* in a male case of HIV chronic infection with imported malaria and latent syphilis by end-point PCR and (**B**) the distance tree of pairwise comparisons of genetic homology and other *P. vivax* strains reported in GenBank^®^.

Confirmation of *P. vivax* was performed by end-point PCR using intergenic variations identified by sequencing of a conserved region of the *18S rRNA* small subunit gene. Genetic fingerprinting of the *Plasmodium* genus was performed according to Rougemont et al., (2004) [13] and compared with the nucleotide sequence database (GenBank^®^) using the heuristic Blast algorithm. The obtained genetic information was used to compare them through multiple alignment using the MUSCLE alignment algorithm [14]. Finally, a distance tree of comparisons was constructed using the sequences deposited in GenBank^®^ using a BLAST tool (NCBI, Bethesda, MD, USA) at the National Center for Biotechnology Information (NCBI). The molecular tools confirmed that the etiology of the imported malaria case was *P. vivax*, which showed genetic similarities to strains identified in the Peruvian Amazon, and it was also similar to clinical strains from Ethiopia. Figure 2A shows the PCR amplification of the molecular target of interest (conserved region of the *18S rRNA* gene) of 160 bp, and Figure 2B shows the distance tree showing the genetic homology of the strain identified in the present case report and other *P. vivax* strains reported in GenBank^®^ [15].

### 2.5. Migratory Route Analysis of the Imported Malaria Case

**Figure 3:**
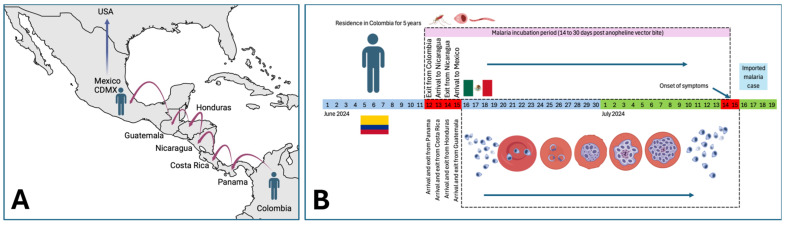
(**A**) Migratory route through Central American countries of the male case of HIV chronic infection with imported malaria and latent syphilis detected in “Clínica de Especialidades Condesa Iztapalapa”. (**B**) Timeline of the migratory route and incubation period of malaria imported to Mexican territory.

According to the migration route shown in Figure 3A, the Venezuelan migrant traveled through five countries before arriving in Mexico City; the chain of countries consisted of Panama, Costa Rica, Nicaragua, Honduras, and Guatemala. Although the migrant is originally from Venezuela, he lived in that country until 2018 before settling in Colombia. In December 2023, he decided to migrate from Colombia to the USA using only land means. The migration route through the five countries is shown in Table 1 and Figure 3A. As shown in Table 1, the migrant’s departure from Colombia was on 12 June 2024, the date considered as the starting point for the epidemiological analysis in the timeline to determine the country where he could have acquired the parasite infection. Given that the migrant traveled through the five countries in four days (on average one day per country) and considering that the incubation period for malaria can be from 14 to 30 days along with the appearance of signs and symptoms and under the background of exposure to mosquito bites during his stay in Panama on 12 June, we speculate that the infection was acquired in that country. Figure 3B shows the epidemiological timeline (in days) for the incubation of imported malaria in the case with HIV chronic infection and latent syphilis.

### 2.6. Antimalarial, Antimicrobial, Antiretroviral Treatment, and Progression

The patient received dual antimalarial treatment (for free and intrahepatic parasites/hypnozoites) until 1 August 2024, due to limited access to the drug. Treatment consisted of 5 chloroquine tablets (150 mg each) plus 2.5 primaquine tablets (15 mg each) on day 1, 2.5 chloroquine tablets plus 2.5 primaquine tablets from day 2 to day 4, and finally 2.5 primaquine tablets from day 5 to day 7. On 5 August 2024, the absence of free parasites was confirmed by thick-drop microscopic analysis, after examination of 500 fields on three slides; however, treatment was continued for eradication of hypnozoites. The reduction in parasitemia was directly related to the reduction in signs and symptoms shown upon admission to the health center (diaphoresis, myalgia, chills, nausea, and fever). Due to reactivity to *T. pallidum*, specific antimicrobial therapy was started, consisting of three doses of benzylpenicillin (2.4 million IU) intramuscularly every 7 days. The patient refers to previous antiretroviral therapy (ART) with a Dolutegravir/Lamivudine/Tenofovir (DTG/3TC/TDF) regimen, which kept him in virological suppression and moderate immunological control. Due to the lack of this ART, the compatible ART was based on Abacavir/Dolutegravir/Lamivudine (ABC/DTG/3TC); however, due to the lack of knowledge of the patient’s immunogenic background (HLA-B* allelic configuration) it was decided to assign ART based on Bictegravir/Emtricitabine/Tenofovir Alafenamide (B/FTC/TAF), which also has no interactions with antimalarial treatment. Subsequent molecular HLA typing studies using sequence-specific oligonucleotides (SSO) showed that the identified HLA-B* allele (HLA-B*44) could not have interfered with the ABC/DTG/3TC ART. Finally, the dosage of the new ART and likely adverse effects are explained to patients. As a result of the comprehensive treatment, a favorable prognosis is expected for the course of his life, with a reserved prognosis for function, but not free of complications.

## 3. Discussion

The increase in migratory mobility in Latin America has brought with it various consequences, including the importation of infectious diseases into Mexico, as well as the challenge of comprehensive healthcare for the migrant population with acquired diseases and/or diseases of origin. To our knowledge, this is the first case report of malaria imported into Mexico in a patient with HIV chronic infection and latent syphilis, which is relevant due to the current migration crisis and the impact it has on the health of people with chronic diseases and other illnesses that they acquire during their migratory journey to the USA. This case highlights the importance of improving epidemiological surveillance of these types of non-endemic diseases through comprehensive care of migrants with comorbidities. This situation shows the vulnerability of patients with HIV chronic infection in a migration situation, especially those without access to ART, as exposure to endemic diseases such as malaria and lack of adequate medical care (including ART) can aggravate their condition, increasing the risk of complications. HIV chronic infection patients with malaria present with complex clinical pictures, as HIV-associated immunosuppression increases susceptibility to co-infections, including bacterial, viral, and fungal infections. We speculate that viral suppression, CD4+ cell counts (which were below minimal limits), medical care, and timely drug treatment were key to avoiding complications in this patient.

Previous work has linked the compromised immune response to HIV chronic infection and malaria complications, which makes it difficult to eliminate the parasite even with antimalarial treatment, leading to disease severity and risk of relapse [16]. In contrast, it has been reported that patients with HIV chronic infection and malaria are at higher risk of developing co-infections such as tuberculosis, making patient management more complex due to drug–drug interactions and the possibility of resistance [17,18]. In the case of co-infections with bacterial STIs, the scenario could be interesting, although to our knowledge there are no reported cases of co-infection of malaria + HIV chronic infection and other STIs, such as latent syphilis. Nonetheless, it is not difficult to speculate that these cases of co-infection with STIs are not identified in health centers, due to situations related to social stigmas and late manifestation of syphilis, among others, leading to underreporting of cases. In a study recently published by “The Lancet Regional Health Americas” and by our working group, the problem of syphilis has been demonstrated in the male population in Latin American countries such as Colombia, Venezuela, and Mexico due to migration and nightclubs for the remunerated sexual activity in border areas [7,18]. A similar case occurs with gonococcal urinary tract infections (gonorrhea), since, in our country, through a national epidemiological study, we demonstrated that this disease continues to be, together with syphilis, the most prevalent STI in the Mexican population [8]. This underlies the importance of comprehensive antimalarial and antimicrobial treatments for STIs in migrant populations, as this limits the spread of pathogens that may have antibiotic resistance determinants, such as those already identified in Latin America [19,20].

Nevertheless, because the antimicrobial treatment administered was based on epidemiology and national regulations, we do not know if the infection was resolved, due to the lack of follow-up to determine whether the case was related to antimicrobial resistance. A relevant aspect is the importance of centers specializing in sexually transmitted infections (STIs) in Mexico, such as the Clínica de Especialidades la Condesa Iztapalapa, which is positioned as a center for the timely detection of diseases imported into Mexico. It is, therefore, necessary for these types of institutions to be able to provide HIV-positive migrants with timely access to ART treatments, as well as STI diagnoses, in order to reduce the burden of disease in the country.

Given that malaria is endemic in several regions of South America, particularly in rural and jungle areas where *P. vivax* transmission is common [21], we speculate that this disease may have been acquired during its migratory path. The epidemiological route, timeline, and dates of migratory transit shown in Figure 3 and Table 1, together with the history of exposure to mosquito bites, suggest that the patient acquired malaria in Panama, a country considered endemic for this disease [22,23].

Conversely, barriers to universal access to ART include lack of resources, stigma and difficulty in integrating into the health systems of the target countries, among others. According to the general health law, Mexico, as a migrant-receiving country, has the responsibility to provide healthcare (including ART), timely detection, and treatment of imported diseases [24]. In the case of migrants, such as the one reported in the present case, lack of access to ART may have increased the risk of developing serious infections and limited treatment options once they arrived at their destination. However, given that he was provided with a new ART regimen in a timely manner and that it did not interact with the antimalarial treatment given, he was able to continue with virological suppression. Importantly, as migration will continue to increase in the coming months or even years, the risk of importing more diseases is latent, which is why it is essential that health professionals are trained to recognize symptoms of rare diseases such as malaria, which are rare in Mexico City where it is not endemic.

Therefore, it is likely that health professionals do not have direct experience in the management of this disease. Nevertheless, in the context of increasing migration, continuous training in tropical and endemic diseases is vital. Continuous training and refresher programs on imported diseases, diagnostic methods, and treatments should be provided to health personnel as this will not only benefit migrants but will also strengthen the capacity of the health system to respond to future health problems. Finally, among the limitations identified in this work is the follow-up of the case, since although the patient had specific treatments, only the absence of the parasite was confirmed, and possible relapses of malaria or complete resolution of syphilis could not be known. Another limitation is the access to cutting-edge diagnostic techniques, since even when molecular tools were used to confirm the *Plasmodium* species, these are not methodologies commonly used in Mexico, which limits the epidemiological investigation of imported cases of malaria. And finally, the training of health personnel, since we mentioned the immediate need to update health professionals in the detection of non-endemic diseases such as malaria.

## 4. Conclusions

The case presented shows the importance of multidisciplinary interaction in addressing the health problems of migrants with diseases acquired during their migratory journey, including chronic and latent infections. The integration of areas such as infectious diseases, parasitology, epidemiology and molecular biology, among others, allowed the identification and timely treatment of malaria, HIV, and syphilis, and we highlight the role of the “Clínica de Especialidades Condesa Iztapalapa” in the detection of STIs and imported diseases.

## Figures and Tables

**Table 1:** The migratory route through the Central American countries (epidemiological arrival and departure dates) of the male case of HIV chronic infection with imported malaria and latent syphilis.

Countries	Arrival Date	Transport	Departure Date	Transport
Colombia	15 December 2023	Land	12 June 2024	Land
Panama	12 June 2024		12 June 2024	
Costa Rica	13 June 2024		13 June 2024	
Nicaragua	13 June 2024		14 June 2024	
Honduras	14 June 2024		14 June 2024	
Guatemala	15 June 2024		15 June 2024	
Mexico	15 June 2024			

## Data Availability

Data will be made available upon reasonable request by accredited researchers.
